# Iodine-123-metaiodobenzylguanidine cardiac SPECT imaging in the qualification of heart failure patients for ICD implantation

**DOI:** 10.1007/s12350-018-1288-6

**Published:** 2018-04-25

**Authors:** Anna Teresińska

**Affiliations:** The Cardinal Stefan Wyszyński Institute of Cardiology, Alpejska 42, 04-628 Warsaw, Poland

Heart failure (HF) is a complex clinical syndrome, with the prevalence of approximately 1-2% of the adult population in developed countries, significantly rising among elderly people.^1^,^2^ The mortality rate of patients with HF remains high despite optimal pharmacological treatment (approximately 50% within 5 years of diagnosis). Sudden cardiac death (SCD), in most cases due to ventricular arrhythmia (VA), accounts for up to 50% of deaths in HF. The risk of SCD is particularly high in patients with reduced left ventricular ejection fraction (LVEF).

Implantable cardioverter defibrillators (ICDs) can abort potentially fatal VAs by way of an electrical shock or anti-tachycardia pacing. The current American College of Cardiology/American Heart Association (ACC/AHA)^1^ and the European Society of Cardiology (ESC)^2^ guidelines, for the management of HF, recommend ICD implantation in primary prevention of SCD in symptomatic patients (NYHA Class II-III), with LVEF ≤ 35%, > 40 days after myocardial infarction, ≥ 3 months on optimal medical therapy, and expected to survive substantially longer with good functional status.

ICDs reduce occurrence of arrhythmic sudden deaths; however, most SCDs occur in patients who do not qualify for ICD implementation according to current criteria. On the other hand, a high percentage of patients with the device implanted on the basis of reduced LVEF and moderate symptoms never suffer an arrhythmia requiring appropriate ICD therapy. Furthermore, post-procedural complication rate accounts for up to 9% during 5.6 years (excluding a high rate of inappropriate shocks leading to worsening quality of life).^3^ Thus, better methods for prediction of SCD risk and qualification for ICD implantation are needed. Suitable patients to investigate are those who already have an ICD implanted - although occurrence of an appropriate ICD therapy does not necessarily mean that they would have experienced SCD if not the device, it is currently an accepted surrogate arrhythmic endpoint.^4^

HF is associated with activation of the sympathetic cardiac nerves. Mechanisms of cardiac arrhythmias are complex and multifactorial, but changes in cardiac adrenergic system (CAS) are the essential components. The state of CAS can be evaluated with I-123-metaiodobenzylguanidine (MIBG), which is a norepinephrine analog and a tracer for sympathetic neuron integrity and function. Therefore, imaging of CAS with MIBG may further refine HF patient selection, beyond LVEF, for ICD implantation.^4^-^6^

Most published studies on the use of MIBG imaging in HF patients is based on measurements from planar images, with cardiac uptake quantified by the heart-to-mediastinum ratio (H/M).^7^ It was demonstrated that decreased values of late H/M from planar imaging can predict potentially fatal arrhythmic events (including resuscitated cardiac arrest, sustained ventricular tachyarrhythmia, or appropriate ICD therapy) as well as cardiac death (including SCD) in patients with HF and thereby help guide the use of ICD.^8^ The ADMIRE-HF trial demonstrated in a large prospective study that the late H/M from planar images was an independent predictor of potentially life-threatening arrhythmic event (AE).^9^ In most of those planar studies, cardiac uptake of MIBG was dichotomized to differentiate high-risk from low-risk populations. To assess the full scope of the prognostic potential of MIBG, in the meta-analysis performed by Verschure et al., the late H/M from planar images was used as a continuous parameter and in multivariate analysis was not an independent predictor for arrhythmias.^10^

SPECT technique had been applied to CAS assessment shortly after MIBG introduction for human heart imaging, as the parameters obtained with planar technique provide only global information while 3D imaging has a potential to evaluate global as well as regional innervation. It has been proposed that patients with MIBG regional defects, especially in areas of preserved perfusion, what has been shown to predispose to denervation super-sensitivity, are especially susceptible to potentially fatal VAs.^11^ For this reason, the results of MIBG SPECT and perfusion SPECT have been often compared by identifying segments with adrenergic/perfusion mismatches.^6^

A few small-cohort studies using semi-quantitative visual scoring techniques suggested clinical utility for SPECT MIBG in assessing arrhythmic risk.^12^-^15^ Arora et al. studied retrospectively 17 patients with ICD implanted 14 ± 11 months earlier. Patients with ICD discharges had higher MIBG defect scores and a higher number of mismatching segments.^12^ Bax et al., in a prospective study of 50 patients with prior MI, showed that the only variable differentiating between positive and negative inducible ventricular tachycardia (VT) in an electrophysiologic study was the late MIBG SPECT results, with a late summed score (LSS) ≥ 37 having a sensitivity of 77% and a specificity of 75% for predicting electrophysiologic results.^13^ Boogers et al. studied prospectively 116 patients before ICD implantation. Over a mean of 23 months, the LSS was an independent predictor of appropriate ICD therapy and patients with LSS > 26 showed significantly more appropriate ICD therapy than patients with a smaller defect.^14^ Marshall et al., in a prospective study of 27 patients referred for ICD in primary prevention, during median follow-up of 16 months showed that patients who experienced a significant AE had higher early SPECT summed scores (ESS) and higher mismatch scores. Optimal threshold for predicting arrhythmias was ≥ 31 for ESS, with a sensitivity of 78% and a specificity of 77%.^15^

All those studies demonstrated (by using binary categorizations of event risk) that the larger the extent of SPECT MIBG abnormality, the greater the likelihood of VT and two of them showed the same also for the innervation/perfusion mismatch score. However, a large international multicenter prospective ADMIRE-HF trial did not show clinical utility for MIBG SPECT in assessing arrhythmic risk during original analysis of the results in 961 patients.^9^ Secondary rigorous analysis of MIBG SPECT and perfusion images from ADMIRE-HF, performed in 471 patients with ischemic HF, also failed to identify the subjects at higher risk of experiencing an AE during 24-month follow-up. On multivariate proportional hazards analysis, the MIBG SPECT score was independently predictive of AEs but the risk decreased with increasing score as the patients with intermediately abnormal SPECT studies had a higher likelihood of AEs compared to patients with extensive abnormalities. Also, the analysis failed to confirm significance of the global innervation/perfusion mismatch score for prediction of AEs.^11^

Whether CAS activity assessed by MIBG scintigraphy could be helpful in selecting patients for prophylactic ICD implantation was evaluated also in the latest prospective multicenter study including 135 stable HF subjects with a median follow-up of 30 months. LSS from MIBG SPECT was not associated with appropriate ICD therapy. Also, late H/M ratio did not show the association, but patients with intermediate ratio were more likely to have appropriate ICD therapy compared to patients with low and high ratios. Post hoc analysis showed that the combination of late H/M ratio and LVEF was significantly associated with freedom of appropriate ICD therapy and it was concluded that (planar) MIBG scintigraphy seems to be helpful in selecting HF subjects who might not benefit from ICD implantation.^16^

In this issue of the Journal, De Vincentis et al. have also addressed the usefulness of cardiac MIBG imaging in predicting AEs in chronic HF patients.^17^ In the prospective single-center study, 170 patients referred for ICD implantation for primary (92%) or secondary prevention were enrolled. Almost 60% of the patients had ischemic HF. All patients underwent early and late planar and SPECT imaging. The primary endpoint was an AE defined as sustained VT, resuscitated cardiac arrest, SCD, or appropriate ICD therapy. The secondary endpoint was appropriate ICD therapy. The median follow-up was nearly 2 years (1-51 months). It has to be pointed out, however, that for appropriate ICD therapy follow-up was short, achieving maximally 1.5 years, as ICD interrogation was performed at 6, 12, and 18 months. Sixty-nine (41%) patients experienced an AE, of these 22 experienced an appropriate ICD therapy. ESS was the only independent predictor of AE. All the planar and SPECT MIBG-derived parameters failed to be independent predictors of appropriate ICD therapy solely. The authors concluded that in their observation no association exists between MIBG scintigraphy-derived parameters and appropriate ICD therapy. On the other hand, the authors observed that there was a ‘bell-shape’ relation between MIBG parameters and AE and appropriate ICD therapy, i.e., those with intermediate MIBG abnormalities tended to be at higher risk of events. The ‘bell-shape’ phenomenon^11^,^16^ has become the center of gravity for the article. However, the results do not confirm this kind of relation between MIBG results and events. Figure 1A,B in the paper shows the relation between H/M categories and the number of events (patients with events). The results however could be plotted as given below (Table). The data do not show that patients with intermediate MIBG abnormalities tended to be at higher risk of events. Actually, the results confirm the ‘more traditional’ tendency observed for another prognostic endpoint for MIBG scintigraphy in patients with CHF; the lower is H/M, the more likely is an AE and appropriate ICD therapy.^10^

**Table:** 

Group of patients	Total number of patients	Number of patients [and proportion] with H/M < 1.2	Number of patients [and proportion] with 1.2 ≤ H/M ≤ 1.6	Number of patients [and proportion] with H/M > 1.6
All enrolled in the study	170	8	111	51
With AE (arrhythmic events)—primary endpoint	69	5 [63%]	47 [42%]	17 [33%]
With appropriate ICD therapy solely—secondary endpoint	22	2 [25%]	15 [14%]	5 [10%]

Accordingly, a figure presenting the relation between H/M categories and the fraction (proportion) of events for different H/M values shall not have a ‘bell-shape’ appearance but rather a ‘stairs-going-down shape.’ Summarizing, the results presented by De Vincentis et al. are not in line with previous findings suggesting that those HF patients with the intermediate MIBG abnormalities tend to be at the highest risk for AEs.^8^,^11^,^16^

In a retrospective European multicenter study performed by Agostini, arrhythmia or SCD occurred in 1% of patients with H/M ≤ 1.45, 12% of patients with intermediate H/M (1.46-2.17) and in 0% of patients with H/M ≥ 2.18.^8^ Travin et al. showed, that the highest proportion of patients with AEs occurred in a group with LSS between 14 and 28 (21.1%) while for LSS < 14 the proportion of patients with AEs was 8.3% and for LSS > 28 it was 8.7%.^11^ Verschure et al. showed that in a group with intermediate standardized late H/M ratio (1.4-2.10), the percentage of patients with appropriate ICD therapy was highest in comparison to percentages in groups with low and high H/M ratios.^16^

In the present study by De Vincentis et al., inter-observer variability of the summed MIBG SPECT scores between the 3 observers was very low: with visual scoring observers were able to adequately overcome possible variation in a uniform way. The authors do not mention the number of SPECT studies that were considered of sub-optimal quality and provided equivocal results.

Although related to some extent, the problems of the image quality and the intra- and inter-observer variability, are different. For example, a group of experienced observers may demonstrate high agreement in rejecting the low-quality images from clinical assessment. The issue of poor quality of MIBG SPECT imaging in HF patients is well recognized based on the physics of I-123 decay and from the bio-kinetics and biodynamics of metaiodobenzylguanidine.^6^,^18^ First, frequent global reduction of cardiac tracer uptake in HF, extracardiac activities equal to or exceeding the activity in the heart walls, and limited spatial resolution of SPECT technique, often result in the images of heart muscle not being distinguishable from the images of the neighboring structures (lungs, liver). Second, scattered photons from those extracardiac organs are added to the images of heart walls because of the construction of collimators (especially LEHR collimators), further deteriorating the differentiability between the heart and the neighboring organ. Third, high-energy photons (1.1% yield of 529-keV gamma rays) penetrating the septa of a collimator decrease the contrast in registered images. In the study of 50 patients with previous myocardial infarction and LV dysfunction, 3 experienced readers considered all MIBG SPECT images as diagnostic and interpretable but only 38%, 88%, and 94%, respectively, to be of optimal quality.^13^ It has been this writer’s center experience with the post-infarction HF patients qualified for ICD that ~ 25% of MIBG SPECT studies were non-diagnostic and in further ~ 25% of studies the assessment was equivocal. SPECT parameters used (LEHR collimators and FBP reconstruction) were similar to the previous studies.

While LEHR collimators are unlikely to be changed into medium energy collimators in MIBG studies (because of better spatial resolution and sensitivity as well as lower encumbrance for SPECT camera for LEHRs), specific methods of iterative reconstruction and image analysis can improve the quality of MIBG SPECT assessment. The latest and the largest study reporting on the potential utility of tomographic MIBG images assessed visually was based on the reevaluation of 621 ischemic HF patients from ADMIRE-HF population.^11^ LEHR collimators were used and optimized iterative reconstruction protocol with deconvolution of septal penetration to correct for image contamination from high-energy I-123 photons was applied. Despite state-of-the-art reconstruction, in 49% of ischemic HF patients the quality of SPECT data was unacceptable or low: 75 patients (12%) had images judged non-diagnostic and in further 231 patients (37%) images were judged diagnostic but of sub-optimal quality. The intra- and inter-observer variability in this report was low.

Improved assessment of low-count MIBG SPECT images has been proposed by creating polar maps of LV activity distribution with the count circumferential profile methods.^19^,^20^ Clements et al. in a group of 961 patients of ADMIRE-HF Study, beyond applying above-mentioned optimized iterative reconstruction protocol, developed a dedicated algorithm of quantitative assessment, self-adopting to the global tracer uptake indicated by H/R, and found only 8 MIBG SPECTs not analyzable (< 1%).^19^ According to Rispler et al., SPECT/CT could be very helpful in creating polar maps of activity distribution within epicardial boundaries of the heart delineated by the CT component, even in cases in which the MIBG uptake is very low.^20^ However, the proposed circumferential approaches do not have the potential of correcting interfering extracardiac MIBG uptake in the generated profiles and maps.

Consequently, the usefulness of MIBG SPECT imaging, with LEHR collimators and available reconstruction and image analysis protocols, is very limited in the qualification of HF patients for ICD and needs wider multicenter discussion on recommended techniques of MIBG imaging in those patients.

Additionally, as was properly recognized in the discussed paper, the limitation of this and many other studies is the heterogeneous HF etiologies of the enrolled patients including both ischemic and non-ischemic HF.^17^ In ADMIRE-HF population, significantly greater and more severe MIBG SPECT abnormalities were shown in ischemic HF patients.^19^ Therefore, further studies are needed to establish the role of MIBG SPECT imaging in qualification of HF patients for ICD implantation in specific subpopulations, particularly in the most numerous population of ischemic HF referred for ICD in primary prevention.

At the moment, global innervation imaging parameters, like H/M ratio, can be used to broadly classify patients at low and high risk.^21^ Late H/M from planar studies is an imaging tool-of-choice for prognostication in HF patients, and it needs further improvement to be used wider. First, the planar technique needs standardization of the results obtainable from different gamma cameras and collimators. Second, the lower threshold for freedom of appropriate ICD therapy could and should be defined on the basis of H/M values for specific HF subpopulations, as indicated in the latest study by Verschure et al., probably in combination with other acknowledged parameters of prognostic value (like LVEF).^16^

In the light of recent studies, the predictors of potentially lethal arrhythmias are not the sole numbers showing quantitatively the denervation defect size.^11^,^16^ These works suggest the relationship between denervation defect size and arrhythmic risk may not be linear and patients with intermediate defects may be at higher arrhythmic risk. Contemporary idea is to study closer heterogeneity of cardiac innervation, as increased heterogeneity provides higher probability of arrhythmias.^18^,^21^ The ability of SPECT to provide regional information not available on planar images remains a driver for efforts to incorporate this procedure into assessments of patients with HF for AE risk.^6^ It is possible, however, that in the future studies on 3D heterogeneity of innervation and innervation/perfusion mismatch, prognostic value of MIBG SPECT over the planar imaging in HF patients will not be proved. Also, superiority of H/M values from SPECT imaging, which was expected to be more exact and reproducible for global innervation evaluation, has not been proved.^22^,^23^ The basic limitation for improving the value of MIBG SPECT could consist in numerous and serious factors degrading MIBG SPECT quality images, difficult to be resolved by hardware and dedicated software development.
